# Fully Phased Sequence of a Diploid Human Genome Determined *de Novo* from the DNA of a Single Individual

**DOI:** 10.1534/g3.119.400995

**Published:** 2020-07-06

**Authors:** llya Soifer, Nicole L. Fong, Nelda Yi, Andrea T. Ireland, Irene Lam, Matthew Sooknah, Jonathan S. Paw, Paul Peluso, Gregory T. Concepcion, David Rank, Alex R. Hastie, Vladimir Jojic, J. Graham Ruby, David Botstein, Margaret A. Roy

**Affiliations:** * Calico Life Sciences LLC, South San Francisco, CA 94080; † Pacific Biosciences, Menlo Park, CA 94025; ‡ Bionano Genomics, San Diego, CA 92121

**Keywords:** Haplotype, Phased genome, Phased sequencing, Chromosome sorting, Human genome

## Abstract

In recent years, improved sequencing technology and computational tools have made *de novo* genome assembly more accessible. Many approaches, however, generate either an unphased or only partially resolved representation of a diploid genome, in which polymorphisms are detected but not assigned to one or the other of the homologous chromosomes. Yet chromosomal phase information is invaluable for the understanding of phenotypic trait inheritance in the cases of compound heterozygosity, allele-specific expression or *cis*-acting variants. Here we use a combination of tools and sequencing technologies to generate a *de novo* diploid assembly of the human primary cell line WI-38. First, data from PacBio single molecule sequencing and Bionano Genomics optical mapping were combined to generate an unphased assembly. Next, 10x Genomics linked reads were combined with the hybrid assembly to generate a partially phased assembly. Lastly, we developed and optimized methods to use short-read (Illumina) sequencing of flow cytometry-sorted metaphase chromosomes to provide phase information. The final genome assembly was almost fully (94%) phased with the addition of approximately 2.5-fold coverage of Illumina data from the sequenced metaphase chromosomes. The diploid nature of the final *de novo* genome assembly improved the resolution of structural variants between the WI-38 genome and the human reference genome. The phased WI-38 sequence data are available for browsing and download at wi38.research.calicolabs.com. Our work shows that assembling a completely phased diploid genome *de novo* from the DNA of a single individual is now readily achievable.

An international effort to sequence the human genome was first seriously proposed in the mid-1980s, and a formal organized effort was undertaken in 1990. Groups at multiple institutions across the world contributed to the project, requiring significant coordination. All the groups used the arduous method of Sanger sequencing large numbers of BAC clones; the sequences were stitched together using physical mapping and from the overlaps in sequence from clones representing adjacent regions of the genome. More than a decade later, when the first draft genome assembly was generated (Lander *et al.* 2001; Venter *et al.* 2001), it was far from being a ‘complete’ telomere-to-telomere sequence and was not phased. The human reference genome sequence has continually been revised and improved since 2001 as DNA sequencing technology has undergone revolutionary change. The “second generation” technologies or “next generation sequencing technologies (NGS)” have been employed, with great success, to fill in gaps in the human reference sequence (International Human Genome Sequencing Consortium 2004; Schneider *et al.* 2017), and to re-sequence individual human genomes in the service of understanding sequence variation (Abecasis *et al.* 2012).

Human reference genome versions (including the current standard reference genome, GRCh38) are a mosaic of sequence data from many (mostly Caucasian) individual diploid humans. One of the major challenges that has remained is to assign all heterozygous polymorphisms to haplotypes, sets of polymorphisms that belong to the same homologous chromosome, often referred to as the problem of “phase”. Phasing information is important in a range of applications. In population genetics, it can be used to impute SNPs and improve the resolution of genotyping (Browning 2008). In quantitative trait mapping, it allows distinguishing between *cis* and *trans* regulation, analysis of allele-specific expression, and mapping interactions between alleles (Benzer 1955; Tewhey *et al.* 2011). In clinical and medical genomics, it can provide insights into cases of compound heterozygosity and large somatic rearrangements (Horton *et al.* 2008; Pyo *et al.* 2010; van de Ven *et al.* 2012).

Multiple strategies were previously proposed to solve the phasing problem. Many of these leverage co-occurrences of variants on the sequenced reads. Additional information is gained from family structure or population data (Wang *et al.* 2008; Song *et al.* 2017; Choi *et al.* 2018; Zhou *et al.* 2018, 2019b, 2019a). Other methods beyond Illumina short read sequencing such as Pacific Biosciences long read sequencing (Pendleton *et al.* 2015), Oxford Nanopore long read sequencing (Bowden *et al.* 2019), 10x Genomics linked read sequencing (Weisenfeld *et al.* 2017), and Bionano Genomics optical mapping (Hastie *et al.* 2017) can phase regions of genomes with enough diversity. While these phased regions are longer than those generated by Illumina short read sequencing they to terminate in regions of low polymorphism or with high repetitive content (*e.g.*, centromeres, p-arms of acrocentric chromosomes, etc.). Other laboratory methods that have been developed include Strand-seq single-cell DNA strand sequencing (Porubský *et al.* 2016; Porubský *et al.* 2017) and Hi-C (Selvaraj *et al.* 2013; Ben-Elazar *et al.* 2016). These methods can generate chromosome-scale haplotypes; however, sophisticated library preparation or expensive high coverage of sequencing are required.

Sequencing individual separated chromosomes was previously proposed by others (Fan *et al.* 2011; Yang *et al.* 2012; Kaper *et al.* 2013; Chu *et al.* 2017). In this approach, when individual chromosomes were sequenced, co-occurrence of SNPs indicates co-residence on the same homolog. However, to our knowledge, this simple, straightforward technique has not been used to completely phase the sequence of entire chromosomes in *de novo* assembled human genomes.

Here we describe the determination of the essentially complete, fully phased genome sequence of a well-studied diploid human fibroblast cell line, WI-38. The WI-38 human diploid cell line was derived from normal embryonic lung tissue (Hayflick and Moorhead 1961). Hayflick (Hayflick 1965) used this cell line in his seminal work showing that cells in culture have a limited lifespan, and showed that cells became senescent after roughly 50 ± 10 divisions . The WI-38 cell line subsequently became the ‘workhorse’ of the pharmaceutical industry, being used for the production of numerous vaccines including adenovirus, hepatitis, herpes zoster, measles, mumps, rabies, rubella and varicella (Olshansky and Hayflick 2017). It is estimated that the use of WI-38-produced vaccines has greatly reduced the tremendous burden of human suffering from multiple diseases: an estimated 198 million cases of disease were prevented or treated in the U.S. alone, and estimated 450,000 U.S deaths were averted, while the numbers world-wide are estimated to be 4.5 billion cases prevented or treated and 10.3 million deaths averted (Olshansky and Hayflick 2017). As historically and economically important as this cell line is, we found no high-quality reference genome in the publicly accessible databases.

We assembled the WI-38 genome *de novo* from data provided by four complementary techniques: Pacific Biosciences (PacBio) RSII SMRT sequencing; 10x Genomics Chromium linked short reads; Bionano Genomics single molecule optical maps; and sparse (0.08-fold coverage/pool) Illumina sequencing of isolated and pooled metaphase chromosomes. Application of the first three technologies to genome assembly required cognate computational methods, most of which are publicly available and well developed (See (Chaisson *et al.* 2015) for a clear and concise review). Combining all four data modalities together and resolving the phases of heterozygous variants on each chromosome required computational methods described herein.

We used the resulting diploid assembly to compare the genome of the WI-38 line with the current human reference genome. One the most important features of the WI-38 line is that it is believed to have remained diploid since it was originally established in 1961. Nearly 60 years later, our karyotyping shows that the WI-38 genome has not acquired major rearrangements such as translocations. More importantly, our *de novo* phased assembly confirms that the genome has in fact remained diploid and retained its heterozygosity throughout.

The resultant phased WI-38 assembly leads us to conclude that something close to the ideal (*i.e.*, determining the diploid human genome sequence directly from a sample of a single individual’s DNA alone) is now well within the reach of individual laboratories with access to the technologies we used. The same ensemble of methods will serve in situations in which no reliable high-quality reference genome sequence is yet available.

## Materials and Methods

### Cell line

**Figure 1:**
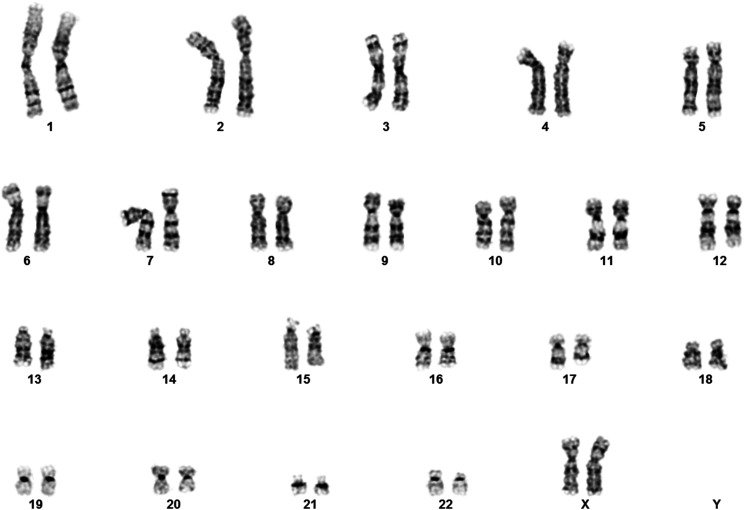
Karyotype analysis of WI-38 PDL 33. Chromosome analysis was performed at a level of 400 bands or greater (Howe *et al.* 2014). Cells at early passage (PDL 33) and at the later passage of PDL 48 (not shown) were observed to be normal human female with 46 chromosomes, two of which were the X chromosome. No major rearrangements were observed.

The untransformed primary cell line WI-38 was obtained from the NIA Aging Cell Culture Repository at the Coriell Institute for Medical Research (catalog AG06814). After expansion of the line in DMEM with 10%FBS (Howe *et al.* 2014), the cells were sent to the Coriell Institute for Medical Research for karyotype analysis to confirm the 2N genotype (Figure 1).

### Single molecule long read sequencing

WI-38 cells were expanded in DMEM with 10%FBS (Howe *et al.* 2014). High molecular weight (HMW) genomic DNA was extracted from the cells using Gentra Puregene kit (Qiagen), and assessed for quality using pulsed-field gel electrophoresis. The HMW DNA was sheared using needles, the g-Tube (Covaris) or the Megaruptor (Diagenode), and 5 ug of sheared gDNA was then used as input into SMRTbell library construction (Pacific Biosciences). The library was size-selected using a BluePippin (Sage Science) electrophoresis unit, and the library fraction greater than 20 kb in length was sequenced on the RSII sequencer (Pacific Biosciences) using P6 polymerase, C4 chemistry and data were collected with 6 hr movies.

### Generation of optical maps

WI-38 nuclei were isolated using isolation buffer from Nuclei Prep Buffer (Zymo Research). Isolated nuclei were embedded into agarose plugs and high molecular weight (HMW) genomic DNA was extracted according to the Bionano Prep Cell Culture DNA Isolation Protocol (Bionano Genomics). HMW DNA was labeled with one of three labeling assays: nicking enzymes Nb.BssS I (NEB) and Nt.BspQI (NEB) were used to label two 300 ng aliquots the HMW DNA according to the Prep Labeling - NLRS Protocol (Bionano Genomics Document 30024H), while a third aliquot (750 ng) of HMW DNA was labeled using the DLE-1 Enzyme assay (Bionano Genomics Document 30206A). Labeled DNA molecules detected using the Saphyr System optical mapper (Bionano Genomics).

### Generation of linked reads

HMW gDNA was extracted from WI-38 cells using Gentra Puregene kit (Qiagen). Linked read libraries were generated using the Chromium Genome V1 kit (10x Genomics) and the Chromium Controller (10x Genomics).

### Generation of Illumina libraries from flow-sorted chromosomes

WI-38 metaphase chromosomes were collected, sorted into 384 well plates and converted into Illumina libraries as follows: WI-38 cells were grown to 60–80% confluency in DMEM + 10% FBS at 37°, 3% O_2_, and cells were stopped in metaphase by treating cultures with 0.1µg Colcemid (15210-040, Gibco) overnight. Mitotic cells were separated from non-mitotic cells by shaking the culture flask and collecting the culture medium, which was now enriched for mitotic cells. Cell culture medium was centrifuged at 200xg for 5 min to pellet mitotic cells. The pellet was washed in once in PBS, resuspended in PBS, and an aliquot was taken for cell counting. Chromosomes were isolated following a modification of (Cram 1990). Modifications included resuspending each pellet in a hypotonic buffer (0.075M KCL, 40uM spermine tetrahydrochloride, 10uM spermidine trihydrochloride) rather than Ohnuki’s hypotonic swelling buffer; and utilizing standard saline flow sheath fluid. Chromosomes were stained for 30 min at room temperature with DAPI (5mg/ml, Invitrogen) and SYBR Safe (1X concentration, Edvotek). Chromosomes were separated by flow-sorting on a BD FACSAria Fusion SORP (15mW 355nm laser, 450/50 bandpass filter; 100mW 488nm laser, 530/30 bandpass filter) using the default “Single-Cell” precision mode on the BD FACSDiva acquisition software with the trigger parameter was set to DAPI detector. Chromosomes were selected by drawing a gate on a bivariate dot plot (DAPI *vs.* SYBR Safe) by selecting for fluorescent signal above background signal. Gated events were subsequently gated by plotting DAPI-H *vs.* DAPI-W and selecting against doublet or aggregated chromosomes. The data acquisition rate was kept under 1000 events/sec for maximum sorting efficiency. Either one or five chromosome(s) were sorted into each well of a 384-well containing 2.5ul Nextera XT TD Buffer (Illumina) and 1.25ul Elution Buffer (Qiagen). Proteinase K (20nL of 20mg/mL, Ambion) was dispensed into each containing sorted chromosomes using the Echo555 Liquid Handler (Labcyte). Libraries were prepared with a 0.25X reaction volume of the Nextera XT Library Kit (Illumina) using 20 PCR cycles. Libraries were pooled and purified with either the Clean and Concentrator-5 (Zymo Research) or with 0.8X AmpureXP beads (Beckman Coulter) SPRI with modified buffer (1X buffer contained 20% PEG 8000, 2.5 M NaCl, 10 mM Tris HCl, 1 mM EDTA, 0.05% Tween 20, pH 8.0 @ 25°). Libraries were sequenced on HiSeq4000 (Illumina) using paired-end 150bp reads with 8bp dual-indexing.

### De novo assembly of the unphased genome

Hybrid scaffolding was performed in four steps. First, we assembled the PacBio long reads using FALCON assembler (Pacific Biosciences version mar2018) with parameters recommended by the vendor for human genome assemblies. Second, consensus optical maps were assembled separately for the optical maps of the three nicking chemistries using Bionano Solve pipeline (v. 3.2.1_04122018). See SI for the parameters. Third, hybrid scaffolding was performed from the PacBio assembly and BssSI and BspQI consensus maps. The conflicts between the assembly and the optical maps were resolved automatically in favor of the optical maps. Finally, another round of scaffolding was performed between the output of the third step and the consensus optical map from DLE-1 chemistry.

### Correcting small and medium-scale assembly errors

To correct the small assembly errors, we aligned linked reads to the PacBio assembly. For technical reasons (since Long Ranger pipeline requires small number of input scaffolds) we first performed a two-enzyme hybrid assembly using Bionano optical maps and after the end of error correction broke the assembly into the contigs and performed hybrid assembly again.

To correct the small assembly errors, we aligned 90x coverage of linked reads to the assembly and called SNVs using Long Ranger (v2.2.2) pipeline. We then selected homozygous (concordant) variants between the assembly and the linked reads and replaced the allele in the assembly with the alternative allele. See SI for details of the method. This process corrected variants up to 10 bp in length.

Correction of the medium-scale assembly errors was done similarly (see SI for the rationale behind this choice). Briefly, 83x coverage of PacBio long reads was aligned to the assembly using NGMLR (v0.2.4) and homozygous SVs were called using PBSV pipeline (v0.1.0 from SMRT Link 5.1.0). We then applied the same script to replace the allele in the assembly with the alternative allele (see SI for more details).

### Initial analysis of chromosome sorted data

To process the chromosome sorted data we first aligned the linked reads to the assembly (using Long Ranger, v2.2.2), called SNPs and phased blocks. At this stage we selected only heterozygous substitutions passing Long Ranger filtering from these calls for simplicity. Reads from chromosome sorting were demultiplexed using bcl2fastq (v2.20.0.422) and aligned to either GRCh38 or to the WI-38 hybrid assembly using BWA-MEM (v0.7.17). We then recalled SNPs using SAMtools (v1.3.1) mpileup using the following parameters: -u -q 10 -t DP,AD and called genotypes using bcftools (v1.3.1) using parameters -c -P 0.5. Individual BCF files from each pool were merged and converted to HDF5 using scikit-allele package (v1.1.8) in Python.

### Assigning scaffolds to linkage group

First, for each sample we determined which scaffolds were enriched in it. This was done in two phases. First, we determined for each sample the threshold between the enriched and non-enriched scaffolds by finding the threshold that maximizes separation between the relative coverages of the two groups (relative coverage is #reads aligning to scaffold per 1 kb / # reads aligning per 1 kb of genome). Second, we calculated Pearson correlation between the enrichment patterns of the scaffolds >5 Mb (*i.e.*, binary vectors with ones for samples enriched for the scaffold) for each scaffold and forming linkage groups between pairs with pairwise correlation > 0.98. This generated (after breaking the chimeric contig) 24 linkage groups (the last group contained a single scaffold that contained a gap > 4.5 Mb and was discarded). Finally, for every enrichment pattern of scaffolds longer than 100 kb we calculated hypergeometric p-value of its overlap with each of the enrichment patterns of the 23 linkage groups, assigning scaffolds with a single p-value < 2^−15^ to the corresponding linkage group. See SI for the detailed description of the process.

### EM algorithm for assigning SNPs to homologs

For simplicity we describe the probabilistic model for a single linkage group, since the phasing is performed on each linkage group separately. Also, for simplicity we performed phasing only on heterozygous SNPs and we excluded SNPs that fall on the regions of the genome that have significantly higher than average coverage of PacBio reads as these were likely unresolved duplications resulting in erroneous SNP calls that seem to be heterozygous.

We define a set of hidden variables $z_{k},\;k=1..K$ where K is the number of SNPs and a set of noise variables ${\text{Φ}}_{l},\;l=1,\ldots L$, where L is the number of wells enriched for a particular linkage group. Specifically, if $g_{lk}$ is the genotype call we have $P\left(g_{lk}=z_{k}\right)={\text{Φ}}_{l}$ and thus the likelihood of the data G is given by

$$P\left(G\right)=\underset{l,k}{\prod }P\left(g_{lk}\text{|}z_{k},\;{\text{Φ}}_{l}\right)={\text{Φ}}_{l}^{\left[g_{lk}=z_{k}\right]}{\left(1-{\text{Φ}}_{l}\right)}^{\left[g_{lk}\neq z_{k}\right]}.$$

Estimation of the parameters using EM approach is given in the SI.

### Generation of phased diploid genome

To generate the phased diploid genome, we aligned PacBio reads to the assembly and called heterozygous structural variants longer than 10 bp using Sniffles pipeline (Sedlazeck *et al.* 2018). We assumed shorter variants were called by Long Ranger and thus already existed in the list of phased variants (Chaisson *et al.* 2019). We used Sniffles rather than PBSV as it was shown to be slightly more sensitive for long structural variants and detects more types of SVs. We then refined insertions and phased the structural variants using CrossStitch pipeline with slight modification that allowed for more careful filtering of the overlapping SVs and phasing of smaller variants (see SI). Finally, we used vcf2diploid (Rozowsky *et al.* 2011) program to create the two versions of the genome.

### Structural variation calls

To call SVs between the reference genome and the diploid assembly, the scaffolds of the diploid assembly were aligned to GRCh38 using Minimap2. Insertions and deletions were called either from breakpoints between the alignments or from the alignment pattern itself. To avoid artifacts, alignments of low quality, alignments completely overlapping alignments of other parts of the assembly were filtered out. We filtered out structural variants in these regions. Inversions were called by looking for pairs of flanking breakpoints, and SVs overlapping gaps were removed. The ends of contigs were highly enriched in structural variants that were difficult to interpret, suggesting that contigs tend to break in highly divergent repetitive regions. SVs were filters, as they likely reflect alignments between highly divergent regions. In addition, regions of significantly higher than average PacBio coverage of reads were defined and were assumed to be regions of unresolved duplications. Finally, regions with multiple close or overlapping SVs as “complex rearrangements” were merged.

### Data availability

The original data (PacBio long reads, 10x Genomics linked reads, chromosome-specific Illumina short reads and Bionano optical maps) and the final assemblies have been deposited in the NCBI BioProject database as accession PRJNA508418. To browse the assembled data, we created a publicly available WI-38 genome browser at wi38.research.calicolabs.com. The browser can be used to view genome assemblies, chromosome-length haplotypes and variant calls. Supplemental material available at figshare: https://doi.org/10.25387/g3.12279338.

## Results

### Determination of WI-38 genome integrity via karyotyping

Before embarking on our deep sequencing efforts, we confirmed, via karyotype analysis, that the WI-38 cell line is a normal, human, diploid and female cell line (see Figure 1).

### Generation of the unphased assembly

**Figure 2:**
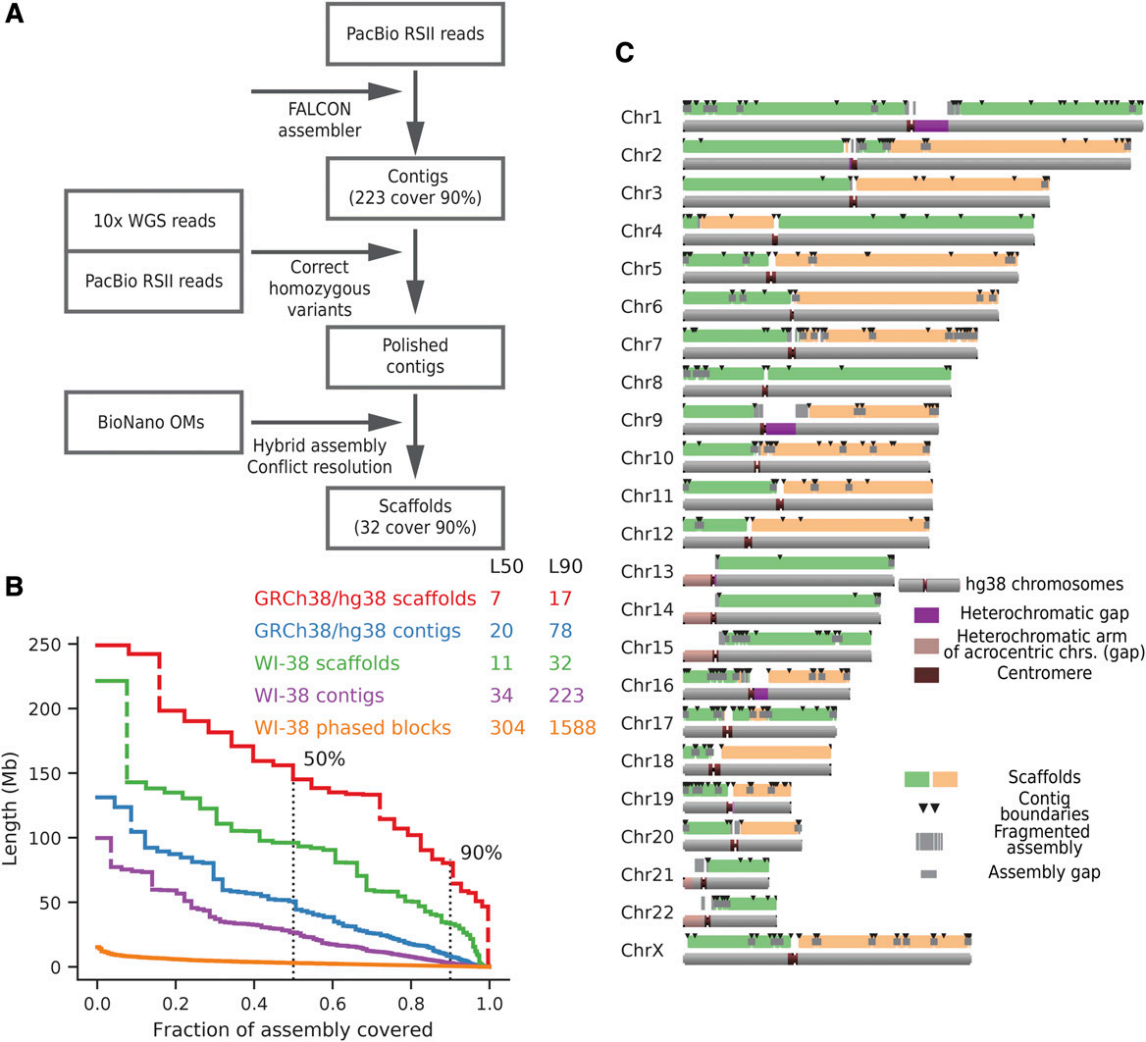
(A) WI-38 hybrid assembly pipeline. PacBio reads were assembled using FALCON, short errors were corrected using discordant calls from linked reads, and larger assembly errors were corrected using homozygous SV calls from PacBio reads. Contigs were then assembled into scaffolds using optical maps and Bionano Solve pipeline. (B) Relative length distribution of contigs, hybrid scaffolds and phased blocks. Note that while length distribution of contigs and scaffolds was similar to the size distribution in the GRCh38 reference genome, phased blocks were of a significantly shorter length scale (C) Hybrid assembly scaffolds. Coverage of human chromosomes by the scaffolds (green and orange bars mark different neighboring scaffolds) and contigs of the hybrid assembly (black lines denote the breakpoints of contigs) shows that most chromosomes arms were resolved by one major scaffold.

**Figure 3:**
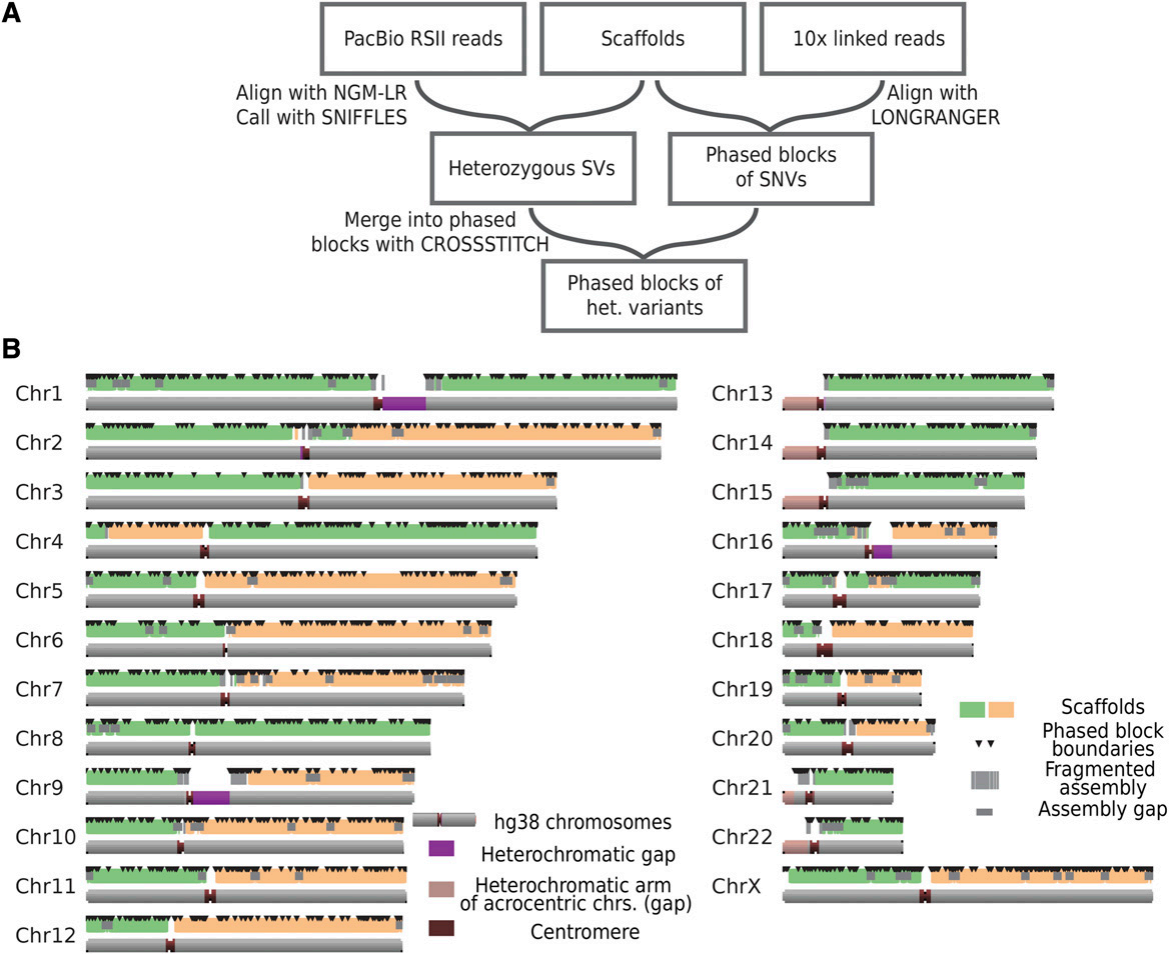
(A) Phasing pipeline. We called SNVs and partially phased them using the 10x Long Ranger pipeline. This generated haplotypes (partially phased blocks) with size distributions as shown below in (B). Heterozygous structural variants were called by aligning all PacBio reads to the hybrid assembly using NGMLR and Sniffles pipelines (Sedlazeck *et al.* 2018). Heterozygous SVs were then assigned to partial phased blocks using CrossStitch pipeline. Individual mitotic chromosomes were then sorted and sequenced to allow for assignment of haplotypes to homologs. (B) Partial phasing does not phase chromosomes completely. Here black triangles denote boundaries between partially phased blocks.

The strategy for generating an unphased assembly is summarized in Figure 2A. First, long single-molecule PacBio reads were assembled into unphased contigs. Second, PacBio contigs were “polished” or corrected with a combination of linked short read data (10x Genomics) that corrected small indel errors and longer raw PacBio reads that corrected larger contig assembly errors. Third, the PacBio contigs were assembled into unphased scaffolds by optical mapping, which resulted in very contiguous but unphased hybrid assembly with scaffolds that traversed entire chromosome arms for most chromosomes. Lastly, these hybrid scaffolds were phased with the addition of SNP data from individual flow-sorted chromosomes and linked reads (Figure 3A).

### Unphased assembly from single-molecule long reads

PacBio RSII single molecule long reads (382 flow cells, roughly 80X coverage) generated total of 13,506,122 long reads with a mean length of 16 kb. At the first step we assembled these reads into contigs using FALCON (Chin *et al.* 2016), one of the standard long-read assembly pipelines. The resulting unphased PacBio assembly contained 1,474 unphased contigs with an N50 of 26.7 Mb (Figure 2B, Table 1) covering over 2.8 Gb of sequence, with the largest 141 of these contigs covering 90% of the genome. Few chimeric PacBio contigs were ultimately resolved by optical mapping (see below), leaving 150 contigs that cover 90% of the genome.

**Table 1:** WI-38 assembly statistics. Statistics from the phased and unphased versions of our WI-38 assembly are compared to the human reference genome GRCh38

	Scaffold Statistics		Contig Statistics		Phase Block Statistics
	GRCh38	WI-38 Hybrid	GRCh38	WI-38 PacBio	WI-38 Hybrid Assembly
Total length	3,099,750,718	2,926,722,723	2,948,627,383	2,856,962,926	2,723,408,086
N50	145,138,636	96,057,442	50,761,348	26,668,154	2,771,792
N90	80,373,285	33,842,722	8,276,615	2,966,364	227,383
Gap count	1,094	12,707	N/A	N/A	N/A
L50	7	11	20	34	304
L90	17	32	78	223	1,588

To evaluate the genic content of this assembly, we estimated the fraction of conserved vertebrate genes present. BUSCO (Simão *et al.* 2015), a pipeline that searches for highly conserved genes in an assembly by a combination of alignment and *de novo* gene annotation. BUSCO detected 94% of the conserved genes in the PacBio assembly, similar to the number found in the version GRCh38.p7 of the human reference genome (Fig. S5).

### Correction of the unphased PacBio assembly

Small assembly errors in the WI-38 PacBio assembly were corrected or ‘polished’ by alignment of 90-fold coverage of 10x Genomics Chromium linked reads to the PacBio assembly and calling variants between the reads and the assembly.

Variant calls from the linked reads are expected to be concordant with one of the alleles in the PacBio assembly (concordant variants), corresponding to the heterozygosity in the WI-38 genome. Variants where both alleles are inconsistent with the assembly (discordant variants) were considered to be PacBio assembly errors. A total of 3,104,497 linked read variants were concordant with the PacBio assembly, while another 1,737,400 variants were discordant, suggesting single-base error rate of approximately 1:2,000bp (Fig.S1A). SNPs constituted the large category of concordant variants (>80%). This rate is in line with the expected heterozygosity of the human genome. Discordant variants (85%) were primarily indels, consistent with the indel rate in PacBio assemblies shown by previous studies (Gordon *et al.* 2016; Seo *et al.* 2016; Wenger *et al.* 2019). We corrected PacBio assembly by replacing the assembly sequence of the discordant variants with one of the alleles called from the linked reads (See Methods and SI).

To evaluate the precision of the unphased PacBio assembly after polishing with linked reads, the occurrence of frameshift mutations caused by indels was determined for a subset of open reading frames (ORFs). Using OrthoDB (Zdobnov *et al.* 2017), a subset of 2,153 human transcripts was selected according to the following criteria: they must exist as a single copy in the reference genome, and they must be represented by only a single annotated isoform in the human RefSeq database (O’Leary *et al.* 2016). This subset of 2,153 human transcripts would theoretically be very unlikely to contain frameshifts.

The unphased and unpolished WI-38 PacBio assembly contained at least one frameshift mutation in 434 of these 2,153 conserved transcripts. After polishing, only eight frameshift mutations remained (Fig. S1B). Upon manual examination, four of these eight frameshifts were located in gaps that were too large to be corrected by the linked read data. Three frameshifts had insufficient read coverage to allow reliable correction. One last frameshift was confirmed by the linked read data and was located near the 3′ end of the ORF sequence; this likely represents true variation in WI-38 genome rather than an error in the assembly (Table S2). Overall, the accuracy of the assembly increased on the order of 100-fold as a result of polishing the PacBio assembly with the linked read data.

Linked reads thus proved sensitive in detection and correction of substitutions and short indels in the assembly. However, short read data were less sensitive in detecting larger errors in the assembly (SI, and see also Chaisson *et al.*, 2019). Errors between 1- bp to ca. 10 kb in length were detected by mapping raw PacBio reads back to the assembly. This resulted in a set of 477 discordant variants containing total of 27.2 kb of either collapsed or expanded sequence (Fig. S2-S4) that belonged predominantly to tandem repetitive areas or low complexity sequence (Fig. S4C). These assembly errors were also repaired.

### Scaffolding of PacBio assembly using optical maps

The corrected PacBio assembly was further scaffolded using optical maps. Three distinct WI-38 optical maps were generated using three different sequence-specific labeling methods at 100X coverage each. Nb.BssSI, Nt.BspQI, and DLE-1 labeling chemistries yielded maps that had contig N50s of 1.8 Mb, 2.1 Mb and 59.5 Mb respectively. These maps were iteratively generated to scaffolds with the unphased PacBio assembly contigs (each iteration resolved any conflicts in favor of the consensus optical maps). The final hybrid assembly from the combination of the PacBio assembly and the optical maps contained 2.9 Gb of sequence with scaffold N50 of 92 Gb (Table 1, Figure 2C). The assembly was highly contiguous, containing 90% of the WI-38 hybrid assembly in only 32 scaffolds.

The unphased hybrid WI-38 assembly was compared to the GRCh38 reference genome (Figure 2C, and Methods). Most of the chromosome arms were resolved into a single scaffold (the major exceptions were the short arms of the 5 acrocentric chromosomes, Figure 2C). Notably, chr1 and chr8 both contained scaffolds that spanned their centromeres. Alignment of the unphased hybrid assembly to the GRCh38 reference genome revealed that both assemblies were generally consistent. A single scaffold that aligned both to chr22 and chr14 was later discovered to be a mis-assembly and was manually corrected (see below and Methods). Major structural differences such as translocations were not observed in the final hybrid assembly, consistent with the WI-38 cell line being a normal primary diploid cell line.

### Phasing of the WI-38 genome and its comparison with the reference genome

Figure 3A shows the strategy that was used to phase the assembly. Specifically, linked reads were first aligned to the assembly to generate haplotypes (phased blocks) of SNPs. Then, heterozygous structural variants were called by aligning the raw PacBio reads to the assembly. Finally, these structural variants were assigned to their linked-read haplotypes by aligning the reads that support them to each of the two haplotypes and choosing the better supporting haplotype. At this stage, most of the variation was assigned to a haplotype of a characteristic size of about 2.8 Mb.

To generate chromosome-length haplotypes, libraries containing individual chromosomes were generated and sparsely sequenced. The principle was to separate individual chromosomes into pools (containing small numbers of chromosomes) so that the likelihood of both homologs being present together in the same pool is low. When these libraries are sequenced, co-occurrence of SNPs in the same pool provides strong evidence of their co-residence on the same haplotype. Integrating assignments from phased blocks allowed imputation of the phasing for SNPs that were not directly observed in the sparse data.

### Phasing using single molecule reads and linked-read data

Linked read data were previously shown to perform well at phasing SNPs due to low error rate and long effective fragment length (Choi *et al.* 2018). We initially phased SNPs using alignment-based Long Ranger pipeline (Zheng *et al.* 2016). Long Ranger generated haplotypes (phased blocks of SNPs) that spanned >2.7 Gb with phased block N50 of about 2.8 Mb (Table 1, Figure 2B, 3B). Phasing the genome with PacBio reads alone was also attempted using the FALCON-Unzip pipeline (Chin *et al.* 2016), but this resulted in significantly more fragmented phased blocks (phased block N50 = 380 Kb).

Linked read-based analyses, however, were not sensitive at discovering long structural variants (Fig. S2A), meaning that many of the heterozygous structural variants in the WI-38 genome were unphased at this step in the assembly phasing process. Since long read-based methods are significantly more sensitive at discovering structural variants, we aligned long reads to the assembly and called structural variants (see Methods and SI). Further, to assign structural variants to the phased blocks, we used a modified CrossStitch pipeline (https://github.com/schatzlab/crossstitch; see SI) that first assigned PacBio reads to the phased block according to the SNPs they span and then assigned structural variants to the phased block according to the assignment of the PacBio reads that span them. This confidently assigned 83% of the structural variants to the phase block (Fig. S7E). In summary, at the end of this step, most of the heterozygous variants (SNVs and SVs) were assigned to a haplotype in partial phased blocks of N50 of ∼2.8 Mb.

### Phasing with flow-sorted chromosomes

Many genome phasing methods fail in regions when heterozygous variants are separated by interspersed large regions of homozygous sequences. Our flow-sorted chromosome method links two heterozygous regions even when interrupted by a long stretch of low heterozygosity; in effect, this method produces longer effective fragments.

**Figure 4:**
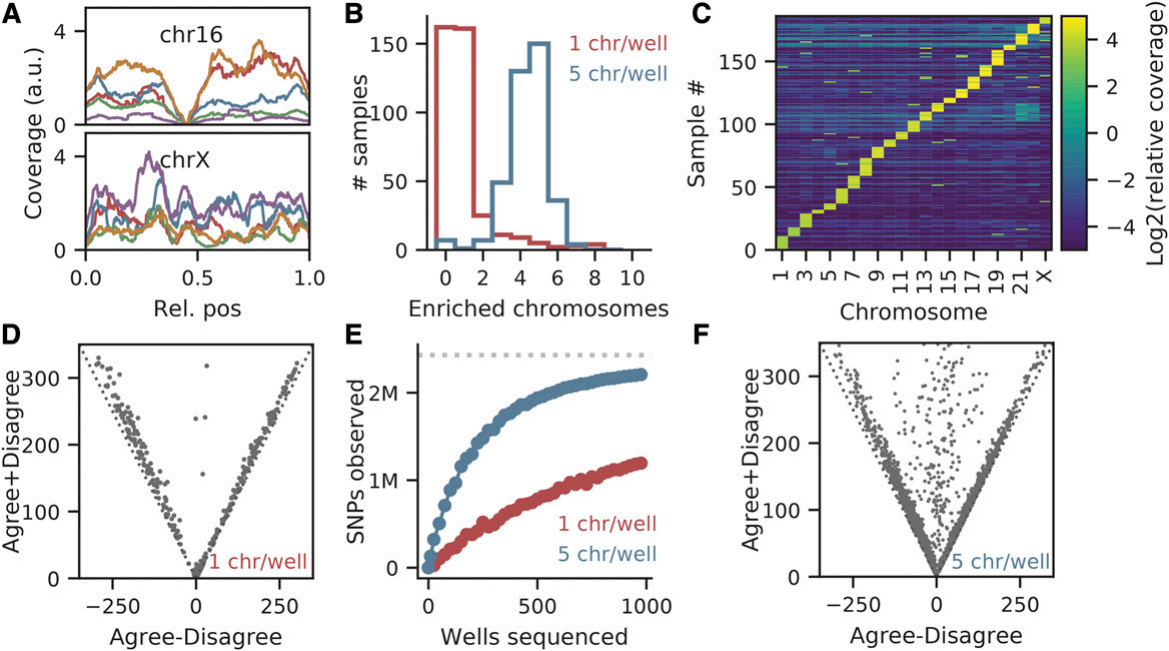
Chromosome sorting isolates individual chromosomes (A) Read coverage. The two panels show the Illumina read coverage from five individual chromosomes mapped across Chr16 (Fig. 4A, upper panel) and ChrX (Fig. 4A, lower panel). Note that the coverage is somewhat uneven on a per Illumina library basis but reasonably evenly distributed in aggregate, except around the centromere for Chr16. (B) Validation of flow-sorting individual chromosomes in 384-well plates. Flow sorting parameters were set so that either one or five separate chromosome events were sorted in wells of a 384-well plate. When mapped to the reference genome, data from plates that targeted one chromosome per well were enriched in reads from one chromosome on average (red line). Wells with 5 chromosome events showed 5 chromosomes on average (blue line). (C) Heatmap of chromosome coverages in each library. Data from single chromosome-sorted libraries were mapped to the reference genome. The X-axis represents the identity of each of the 23 chromosomes while the Y-axis represents the data from each chromosome (data from one chromosome sorted per well in a 384-well plate). As expected, the heatmap shows that reads from each well mapped predominantly to only one chromosome. (D) Pairs of libraries enriched for the same chromosome either agree or disagree on the sampled SNPs. Every point corresponds to a pair of libraries enriched for the same chromosome, Dotted line shows the expected result if SNPs come from the either the same or opposite homologs. (E) Sequencing depth *vs.* SNP content. As expected, sequencing a greater number of wells leads to a greater number of SNPs in the dataset. This occurred whether one chromosome per well (red dots) or five chromosomes per well (blue dots) were sorted and sequenced. Note significant improvement in SNP detection when pooling five chromosomes per well (F) Data from wells containing five chromosomes still tended to always agree or always disagree on the sampled SNPs. Notation as in (D). But with five chromosomes per well, the number of wells with both haplotypes of a chromosome is higher. If these data are ignored the overall sequencing yield of the number of unique chromosomes per well is still greater than with one chromosome per well.

WI-38 mitotic chromosomes were isolated, stained and then sorted on a flow cytometer (Methods). Individual chromosomes appeared as discrete flow events and clustered into clouds based on their size. Individual chromosomes were randomly sorted into 384-well plates. Barcoded short read libraries were created from each well using a tagmentation approach (Adey *et al.* 2010), then pooled and sequenced on an Illumina HiSeq 4000 (Methods). Most of the reads mapped to the human genome in a relatively uniform manner (Figure 4A). We observed, however, a high duplication rate (∼5% of reads were unique) as expected from low input tagmentation libraries (Table S4). This creates an inefficiency that can be avoided by pooling several chromosomes per well (see below).

Next, read coverage per well (normalized to length) of each chromosome was calculated. In 70% of wells, there was a small subset of chromosomes with ∼100-fold more reads than the other chromosomes (Fig. S6A-B, Figure 4B), as expected. In most wells, a single chromosome was strongly enriched (Figure 4C). Thus ∼70% of the wells contained one (or very few) chromosomes.

Finally, to examine the utility of the single-chromosome data for its use as a genome phasing method, the reads were aligned to the reference genome GRCh38 and the SNPs were called from the alignments (SI). Greater than 90% of the SNPs were homozygous (on average 7,791 SNPs only were observed in data from each well). Most importantly, if two compared wells were enriched for the same chromosome identity (for example, chr1), they tended to either strongly agree or disagree on the alleles of the SNPs of that chromosome (Figure 4D). This is consistent with each well containing one homolog but not both. A small fraction of pairs of wells agreed/disagreed on roughly 50% of alleles, which is consistent with one of the wells containing two homologs of the same chromosome.

### Pooled sorting improves efficiency of phasing

Creation of libraries from low input DNA amounts is challenging; the data are almost always plagued by strong sequencing biases and low library complexity (Navin *et al.* 2011; Fu *et al.* 2015). Consistent with these previous studies, the libraries had low complexity and a high fragment duplication rate of 95% (Table S4). Thus, the number of SNPs that were detected in each well was low and we estimated that over 2,500 wells would be necessary to cover >95% of SNPs assuming random sampling (Figure 4E). We therefore sought a way to increase complexity of the sequencing libraries to reduce the number of wells required.

We reasoned that if we sort low numbers of random chromosomes into a well, the probability that two different homologs of the same chromosome will be sorted together will still be low. Therefore, we prepared libraries by sorting five chromosomes into each well. In this case, approximately 20% of wells are expected to contain both alternate homologs. If any well contains data from both haplotypes, the data from that specific well is removed.

When evaluated, the dataset from sorting five chromosomes per well did indeed contain on average 4.36 ± 1.12 chromosomes per well (Figure 4B). Additionally, the average number of unique reads increased significantly to 17% unique reads (*vs.* 5% seen in the data from one chromosome per well) (Table S6). Pairs of wells in this batch almost always strongly agreed or strongly disagreed on which of the two chromosome alleles (SNP sets) were enriched in a well, consistent with low probability of sorting two homologs per well (Figure 4F). More importantly, the new set of libraries significantly increased the number of SNPs observed in a well (16,690 ± 5577 on average) both due to the increased library complexity (Figure 6) and increased probability of the library to be enriched for chromosome (Figure 4E).

### Phasing of de novo assembled genome scaffolds

To phase the hybrid assembly, the data from the sorted chromosomes was aligned and scaffold enrichment in each library was calculated as above. First, we sought to assign the hybrid assembly scaffolds to the chromosomes. To accomplish this, a $N_{\dot{scaffolds}}\times N_{samples}$ binary matrix was generated with 1 in position (i,j) if scaffold i was enriched in a well j. Clustering the rows of this matrix revealed 22 co-occurrence patterns (Fig. S7B).

**Figure 5:**
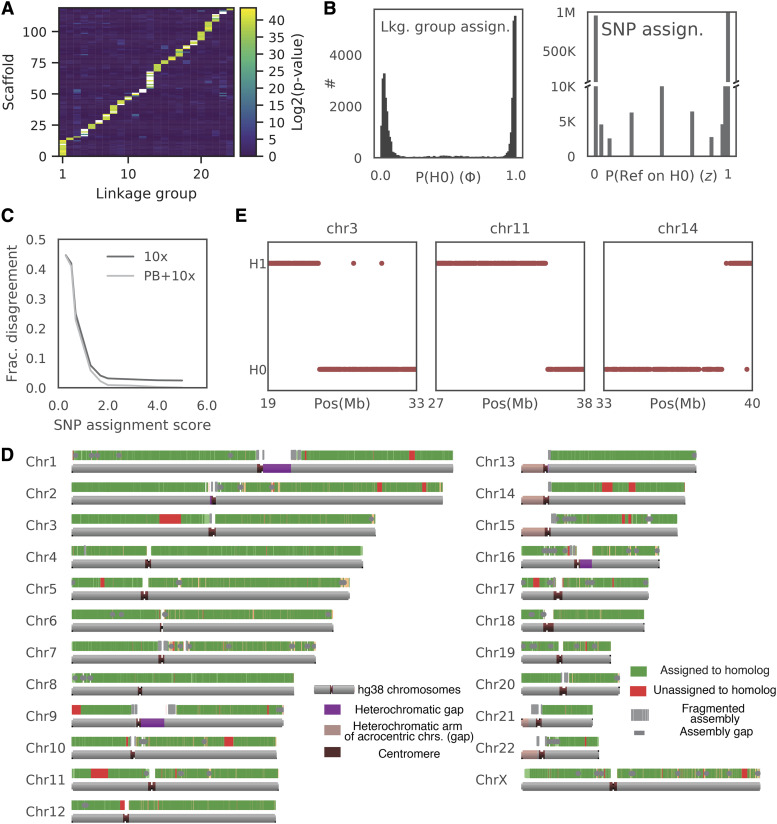
Phased WI-38 Assembly. Combining SNP phasing from flow-sorted chromosomes with linked-read phased blocks allows accurate assignment of >90% of polymorphisms to their respective homolog. (A) Assignment of scaffolds longer than 100 kb to the linkage group. Heatmap shows probability that a given scaffold belongs to one of the linkage groups. Only scaffolds with a single possible linkage group are shown (see Fig. S7B-C for all scaffolds). (B) Phase of the well (Φ) and homolog assignment of the SNP (z) were confidently estimated using EM. Left: Histogram of probabilities that the well is enriched for a certain chromosome contains homolog zero *vs.* homolog 1 for all (well, enriched chromosome) pairs. Right: Histogram of assignment confidence to a homolog for each SNP. (C) Phase estimates were highly concordant between chromosome-sorted data and the partial phasing. We binned SNPs according to their assignment score -log10(min(z,1-z)) and calculated the fraction of those that disagree with results of partial phasing by linked reads (black line) or of a consensus of linked-reads-derived phased blocks (haplotypes) and FALCON-unzip-derived haplotypes. Note that for over 90% of SNPs with assignment confidence of > 98% there is < 2% discrepancy rate with partial phasing (if the phasing is consistent between 10x and PacBio). (D) Results of assignment of partially phased blocks to homologs. Green - assigned regions, Red - unassigned blocks. Note that most of the unassigned blocks were not assigned due to a phase switch in the middle of the phased block (see(E)). (E) Patterns of agreement. Comparison of the chromosome sorting assignment to the partial phasing in the three largest unassigned phased blocks. Note an apparent breakpoint in homolog assignment that is consistent with a mistake of Longer Ranger pipeline).

Further analysis revealed one particular scaffold where reads aligning to the first 35 Mb were enriched in one set of wells, while reads aligning to the remaining 87 Mb were enriched in a different set of wells. This data suggested a chimeric scaffold that links together sequences on two chromosomes (Fig. S7A). We therefore determined the most likely merge point and used the chromosome sorted data to break the chimeric scaffold. The enrichment pattern of the resulting scaffolds revealed 23 co-occurrence (linkage) groups that were consistent with the chromosomes that the scaffolds aligned to. Thus, the sorted chromosome data were useful not only for phasing, but also to correct and improve the assembly by assigning scaffolds to chromosomes. We then assigned an additional 77 smaller scaffolds to the linkage groups using a hypergeometric test (see Methods). In total, 2.88 Gb (98.2%) of the hybrid assembly was assigned to linkage groups (Figure 5A, S7B-C).

**Figure 6:**
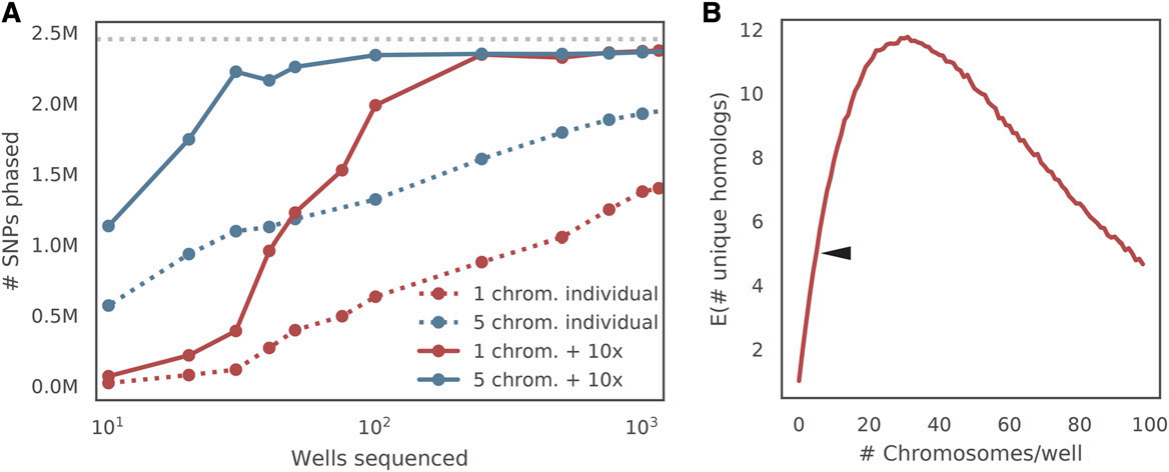
Optimization of data yield from chromosome sorting (A) Relationship between number of wells sequenced and number of SNPs phased correctly. Using more than one chromosome per well increases the efficiency of sorting and library production. The solid lines show the number of SNPs correctly assigned to homolog if phasing is done using partially phased linked-read blocks, while the dashed lines show the number of SNPs correctly assigned to homolog if phasing is done independently. Red and blue lines show data collected from one chromosome or five chromosomes per well respectively. Note that the correctness of the SNP assignment is determined by using the maximal number of wells sequenced. (B) The number of unique flow-sorted homologs per well. Based on simulations, the optimal data yield (number of unique chromosomes per well) is about 25 chromosomes per well.

Next, the chromosome sorted data were used to assign the observed SNPs to their respective homologs. For simplicity, we only phased substitutions, using the partially phased blocks to phase other types of mutations (see below). This process achieved three goals. First, phase assignments of a SNP from different wells were aggregated (some SNP calls were incorrect due to a low level of background noise). Second, wells containing both homologs of a chromosome were filtered out. Third, information from all SNPs in a partially phased block was aggregated so that partially phased blocks were assigned to homologs. 

In order to assign observed SNPs to homologs, we formulated a generative model that was fit to the data under the assumption that every SNP belongs to either homolog 0 or homolog 1, and that for each pair *l* (per well, enriched chromosome (linkage group) *i*), the enriched chromosome has a constant probability ${\text{Φ}}_{i}$ to produce a SNP from homolog 0 (*e.g.*, if ${\text{Φ}}_{i}∼1$, then the well is enriched with homolog 0, if ${\text{Φ}}_{i}∼0.5$ - the well contains both homologs etc.). More formally, we defined a set of hidden variables $z_{k},\;k=1..K$ where K is the number of SNPs and a set of parameters ${\text{Φ}}_{l},\;l=1,\ldots L$, where L is the number of wells enriched for a particular chromosome. Specifically, if $g_{lk}$ is the genotype call, $P\left(g_{lk}=z_{k}\right)={\text{Φ}}_{l}$ and thus the likelihood of the genotype calls G is given by

$$P\left(G\right)=\underset{l,k}{\prod }P\left(g_{lk}\text{|}z_{k},\;{\text{Φ}}_{l}\right)={\text{Φ}}_{l}^{\left[g_{lk}=z_{k}\right]}{\left(1-{\text{Φ}}_{l}\right)}^{\left[g_{lk}\neq z_{k}\right]}.$$

As described in Materials and Methods and SI we used an EM (Expectation–Maximization) algorithm to fit the set of ${\text{Φ}}_{l}$ and $z_{k}$ independently for each chromosome. Homolog assignments ${\text{Φ}}_{l}$ were clearly bimodal around 0 and 1 for all linkage groups, with a small fraction of wells estimated to contain both homologs (Figure 5B, left). SNP allele assignments to the homologs were also bimodal, with > 80% of SNPs assigned to a homolog with <2% estimated error rate (Figure 5B, right). Consistent with our error estimation, assignments of SNPs to homolog with error probability of <2% differed from the phasing derived from consensus between FALCON-Unzip and Long Ranger in less than 2% of cases (Figure 5C).

High congruence between the chromosome-sorting phasing and partial phasing allowed us to impute phase for unobserved or low-confidence SNPs by assigning partially phased blocks to homologs. This had the added benefit that it further reduced the number of sequencing libraries required to phase the hybrid assembly. Within each phased block, SNPs that were phased in chromosome sorted data with assignment probability $z_{k}<0.02$ or $z_{k}>0.98$ were selected and phased blocks were assigned to a homolog by majority vote. As a result of this process, over 90% of all heterozygous variants belonged to phased blocks that were assigned to a homolog with expected error rate of < 2%, and the corresponding phased blocks spanned 94% of the final hybrid assembly (Figure 5D, Fig. S7D). The phased blocks that were not assigned confidently contained a phase switch relative to chromosome sorted data (red areas on Figure 5D-E). Since the homolog assignment of SNPs in chromosome sorting data are independent between SNPs, this error pattern is more consistent with phasing errors in the Long Ranger pipeline, rather than a mistake in chromosome sorted phasing. Thus, data from flow-sorted chromosomes can repair at least some phase switches in an assembly, but further analysis of this method was not pursued.

### Optimizing the number of chromosomes per well for phasing

After generating a large dataset from flow-sorted chromosomes, we realized that a genome could be fully phased with a smaller dataset. Specifically, when five chromosomes per well were used rather than one chromosome per well, only 30 wells were required to achieve the same phasing accuracy as 1,000 wells when using partially phased blocks to impute undetected SNPs in the phased block (Figure 4F). As discussed above, having five chromosomes per well was clearly superior in producing less sequence bias and a lower read duplication ratio; ∼300 libraries with 1 chromosome per well were needed to achieve the same phasing efficiency.

Pooling multiple chromosomes in a well was clearly beneficial for optimal data yield. We sought to determine the most efficient number of chromosomes in the pool by finding the optimal balance between the number of chromosomes sorted per well and the average number of wells containing both haplotypes of any particular chromosome. This was determined through a simulation of a random selection of chromosomes per well with different pooling sizes. As expected, the simulation resulted in non-monotonic behavior. Roughly 25 chromosomes per well was optimal, producing on average 12 unique homologs with the 13 chromosomes contains both haplotypes in that well. Thus, the efficiency of phasing using chromosome sorting could theoretically be improved further by pooling 25 sorted chromosomes per well, although we did not test this hypothesis. Notably, the existing statistical framework should easily allow for this modification.

### Structural variation in WI-38 genome

SVs were determined from a diploid version of the hybrid genome that was generated using assignment of the phased blocks to homologs from chromosome sorting (see SI). The first step in SV determination involved using the whole genome aligner Minimap2 (Li 2018) to align the diploid version of the WI-38 genome to the GRCh38 reference genome. Most WI-38 hybrid assembly scaffolds aligned to the reference genome, yet a subset of 323 scaffolds longer than 1,000 bp containing 4.3 Mb did not align. Most of these unaligned sequences were tandem repeats (78%) or other types of repeats (63%) (Tables S4). The longest unaligned block was 71,327 bp long. 153 of the 323 unaligned sequences did align to the recent set of pan-genome sequences missing from the reference genome suggesting that this set of 153 scaffolds represents common sequences missing from the GRCh38, rather than being rare structural variants (Sherman *et al.* 2019).

**Figure 7:**
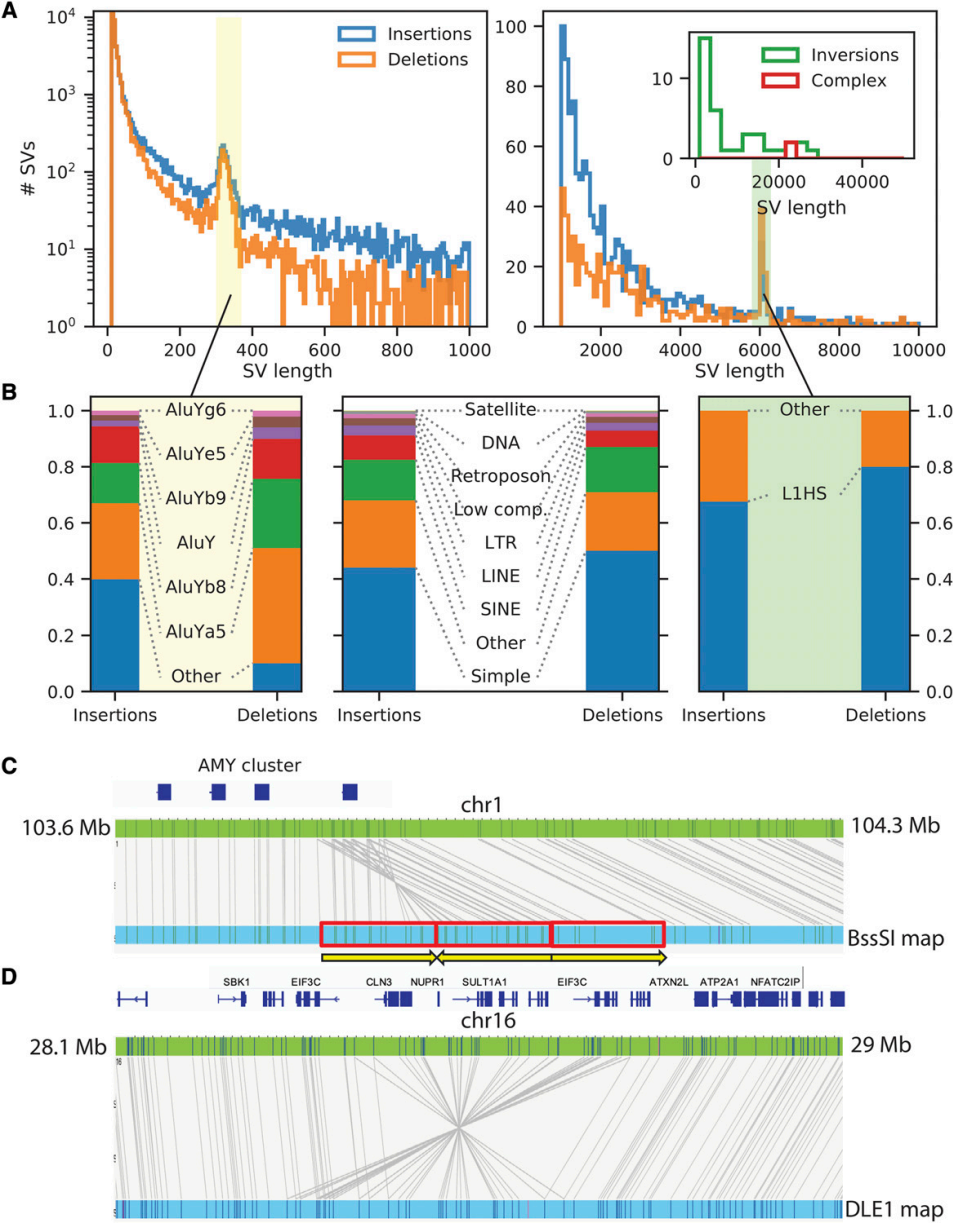
Structural variation between the WI-38 and the reference genome (GRCh38). (A) Length distributions of structural variants. WI-38 SVs were compared of the GRCh38 reference genome and the histograms of lengths of insertions (blue), deletions (orange), inversions (green) and complex SVs (red) are shown. As expected, SVs of ∼300 bp were mostly comprised of AluY TEs (B, left panel) and those of ∼6 kb were highly enriched with L1HS TEs (B, right panel). (B) Structural variants were highly enriched with repeats and transposable elements. Bar charts showing repeat type compositions of SVs. Left panel – when focusing on SVs of ∼300 bp (Other – non-AluY), Right panel – when focusing on SVs of ∼6 kb (Other – non-LINE). Central panel – all SVs (Other – non-repeats). (C, D) Bionano optical maps provide additional interpretation of structural variants. Examples of structural variants identified by in hybrid assembly as “complex” and by interpreted by Bionano optical as inverted duplication (C) and inversion (D). Complex rearrangement on Chromosome 16. Alignment of the DLE-1 optical map (blue) shows a large SV relative to GRCh38 (green).

The next step in determining structural variants involved calling the breakpoints between the alignments of the same WI-38 contig or from the gaps and insertions that the aligner introduced when comparing to GRCh38. We identified 107,287 high confidence SVs that were longer than 10 bp, of which a subset of 43,723 were homozygous and a subset of 63,564 were heterozygous (Figure 7A; see Methods and Fig. S8A-B). Most of the structural variants discovered were short insertions and deletions, although we also found several dozen short inversions and more complex rearrangements. Roughly 50% of structural variants occurred in simple repeat areas, while the remaining 25% were insertions or deletions of SINEs and LINEs (Figure 7B). The number of SVs as a function of size declined approximately exponentially, with peaks at about 300 bp (loss or gain of SINEs, mostly AluY) and 6 kb (loss or gain of L1HS elements) consistent with previous findings (Figure 7B, Table S8, S9).

The ability to call large SVs decreased as the length of the structural variant approached the length of a contig. To determine SVs of larger size, the optical maps from the three labeling chemistries were compared to GRCh38. Bionano optical maps improved longer SV calls in the WI-38 genome. The maps provided better interpretation of structural variants when rearrangements were complicated. For example, the optical map data showed an inverted duplication in the *AMY* region on chromosome 1 (Figure 7C, Fig. S9A), and a heterozygous inversion on chromosome 16 (Figure 7D, Fig. S9B). These two regions were interpreted as complex re-arrangements when the PacBio assembly sequence scaffolds were aligned to GRCh38. The optical mapping improved sensitivity and facilitated interpretation of structural variants even with in highly contiguous scaffolds.

## Discussion

Using only two sequencing methods now generally available, 90% of the unphased hybrid genome is covered by only 32 scaffolds which were assembled from 223 contigs. This high-quality assembly was accomplished by using DNA from only one single individual, without the need for cloning into BACs. By way of comparison, 90% of the current reference genome GRCh38, is covered by the 17 longest scaffolds (*cf*. the bottom row of Table 1) but it is derived from a panel of diverse individuals and relied heavily on the use of BAC clones.

### Phasing provides continuity

Although GRCh38 is designated as *the* human reference genome, it was in fact assembled from multiple individuals. As such, it cannot be phased. Conversely, our *de novo* sequence is derived from a single human cell line. We were able to phase our *de novo* sequence fully using a combination of four genomic technologies and computational pipelines that assigned ca. 90% of all polymorphisms in the dataset. The final assembly has phased continuity of two haplotypes completely across each of the chromosomes, and not just in multiple haplotype blocks within a chromosome. In other words, phasing connects, in a genetically significant way, sequences that span gaps that cannot be handled by existing sequencing technology. Notably, the ability to phase at this scale allows for determination of long-distance genetic linkage, even across regions such as heterochromatin that lie in sequence gaps, (*e.g.*, centromeres and other megabase-sized repeat regions). It is for this reason that we regard our *de novo* WI-38 genome to be a “complete” human sequence. The phased WI-38 sequence data are available for browsing and download at wi38.research.calicolabs.com.

### Phasing of variants provides genetically useful information

Whereas re-sequencing of a single individual’s genome can provide a catalog of sequence variants, a phased *de novo* hybrid assembly provides much more information than can be obtained by “re-sequencing” or aligning reads to a reference genome. The determination of whether two polymorphisms lie *cis* or *trans* to each other is often a critical feature in assessing their likely biological effect. The presence of two potentially mutations in a coding sequence could result in little or no phenotype if they are *cis* to each other, while they might cause loss of function if they are *trans* (Benzer 1955), forming a compound heterozygote. In non-coding regions, a polymorphism thought to encode an enhancer would be expected to affect alleles that lie in *cis* but not in *trans* to the functional sequence it is thought to control. Similarly, an unphased assembly represents only partial picture of the SVs. The phased WI-38 genome contained many heterozygous structural variants, including deletions and inversions that are missed in an unphased assembly.

Multiple methods were previously developed that allow for long range phasing they require either expensive sequencing (Hi-C), or complicated library preparation techniques (Strand-Seq), the availability of parental sequences (trios) or extensive population data.

### Leveraging diverse genomic technologies

While genomic technology has improved since the publication of the first draft sequence of the human genome in 2003, the advancement in instrumentation and computation has been particularly rapid in recent years, finally arriving in a state where high quality *de novo* assembly and phasing of large genomes can become more commonplace. Here we combined instrumentation and computation to produce our phased *de novo* genome. Data were generated from the PacBio RSII instrument to provide long read sequencing, the 10x Genomics Chromium platform to provide Illumina linked-reads; Bionano Genomics Saphyr generated optical mapss, and Illumina HiSeq instrument was used to sequence flow-sorted metaphase chromosomes. The specific technology we used herein have already changed since we generated the data; we can foresee many further additional improvements and simplifications. We used PacBio RSII to generate the PacBio data; this instrument is now obsolete and PacBio released 2 new instruments since we undertook this project. We used version 1 of the 10x Genomics linked read chemistry; 10x subsequently released version 2 that significantly improved the quality of phasing (unfortunately, 10x Genomics recently announced they will no longer manufacture the linked read chemistry).

For this study we flow-sorted either one or five chromosomes per well of a 384-well plate and created barcoded libraries in each well. Moreover, our simulations suggest that sorting approximately 25 chromosomes per well will decrease the sequencing and library-preparation requirements even further.

Chromosome sequencing is relatively affordable and requires usage of widely-available laboratory techniques. Metaphase chromosomes are generated by essentially the same technique that has been used for karyotyping over the last six decades. Sequencing libraries are prepared in 384-well plates with the addition of 1/4 volume Nextera library preparation reagents per well. Only 30 wells are required to phase an entire human genome when five chromosomes per well were sorted by flow-cytometry into each well. This result required use of approximately 2.5-fold genome coverage on an Illumina sequencer, which is negligible relative to the sequencing used to generate partially phased data. Moreover, our simulations suggest that sorting up to 25 chromosomes per well will require fewer libraries and will reduce the library prep cost further. Notably, an efficient use of chromosome sorting requires a source of partially phased data that can be generated by numerous methods.

Future studies in our group will focus on sequencing chromosomes after barcoding them in microfluidic droplets or by combinatorial barcoding approaches, greatly increasing ease of use and making the technique available to labs without access to a flow-sorting instrument. The continued evolution in genomics technology from instrument vendors will enable *de novo* assembly to become increasingly available to investigators outside large sequencing centers.
